# Echocardiographic Evaluation after Transcatheter Aortic Valve Implantation: A Comprehensive Review

**DOI:** 10.3390/life13051079

**Published:** 2023-04-24

**Authors:** Domenico Angellotti, Rachele Manzo, Domenico Simone Castiello, Maddalena Immobile Molaro, Andrea Mariani, Cristina Iapicca, Dalila Nappa, Fiorenzo Simonetti, Marisa Avvedimento, Attilio Leone, Mario Enrico Canonico, Carmen Anna Maria Spaccarotella, Anna Franzone, Federica Ilardi, Giovanni Esposito, Raffaele Piccolo

**Affiliations:** Department of Advanced Biomedical Sciences, University of Naples Federico II, 80131 Naples, Italy

**Keywords:** echocardiography, follow-up, TAVI

## Abstract

Transcatheter aortic valve implantation (TAVI) is an increasingly popular treatment option for patients with severe aortic stenosis. Recent advancements in technology and imaging tools have significantly contributed to the success of TAVI procedures. Echocardiography plays a pivotal role in the evaluation of TAVI patients, both before and after the procedure. This review aims to provide an overview of the most recent technical advancements in echocardiography and their use in the follow-up of TAVI patients. In particular, the focus will be on the examination of the influence of TAVI on left and right ventricular function, which is frequently accompanied by other structural and functional alterations. Echocardiography has proven to be key also in detecting valve deterioration during extended follow-up. This review will provide valuable insights into the technical advancements in echocardiography and their role in the follow-up of TAVI patients.

## 1. Role of Echocardiography in TAVI Patients

Echocardiography plays a key role in the evaluation of patients undergoing transcatheter aortic valve implantation (TAVI) because it is essential for determining eligibility for the procedure and evaluating its efficacy during the follow-up. Transthoracic echocardiography (TTE) enables accurate noninvasive evaluation of valve anatomy and hemodynamics, as well as of cardiac chambers function and morphology prior to intervention. Current guidelines recommend echocardiographic follow-up after TAVI before hospital discharge or within thirty days, six months, one year, and annually thereafter [1]. A comprehensive echocardiographic examination should always include information on transcatheter heart valve (THV) function and should immediately recognize possible prosthesis dysfunction. Moreover, aging is associated with altered left ventricle (LV) diastolic filling and LV hypertrophy. Consequently, the elderly status of TAVI patients, along with additional comorbidities, might negatively affect LV structural and functional recovery after the procedure. Therefore, a complete evaluation of TAVI-induced changes in the LV and right ventricle (RV) function, as well as in the degree of mitral regurgitation (MR) severity, is essential for providing prognostic data and defining the correct therapeutic strategy.

## 2. Impact of TAVI on Cardiac Function and Structure

TTE is the most commonly used method for evaluating LV structural and functional changes during the pre- and post-procedural stages of TAVI. The availability of advanced echocardiographic techniques has allowed for a more thorough assessment of the impact of TAVI on the left heart chambers and concomitant heart valve disorders.

### 2.1. TAVI and Reverse Remodeling

LV pressure unloading following TAVI is a significant driver for LV mass (LVM) regression [2]. Moreover, because TAVI reduces LV wall stress and intra-cavitary pressures, an improvement in diastolic subendocardial perfusion resulting in enhanced LV longitudinal function can be observed shortly after the procedure [3]. Consequently, when the reason for LV dysfunction is the afterload mismatch rather than irreversible myocardial damage (due to fibrosis or coexisting coronary artery disease), functional improvement is anticipated almost immediately following the procedure. Reverse remodeling is a continuous process that occurs over time, with the largest LVM index (LVMi) regression occurring during the first year but still present at a three-year follow-up, with comparable effects in both sexes [4]. An early decrease in LV mass may be due to the regression of myocyte hypertrophy, whereas a late LV remodeling may be due to the regression of fibrosis [3]. Puls et al. demonstrated that myocardial fibrosis was associated with a higher extent of baseline pathological LV remodeling (higher LVMi and larger left ventricle) and with delayed but not inhibited reverse remodeling at a six-month follow-up [5]. Baseline LVMi, relative wall thickness, and moderate or severe aortic regurgitation (AR) at 30 days were independently associated with LVMi regression at one year, with AR being associated with a smaller LVMi decrease [4]. Inconsistent data exist regarding the relationship between factors linked to increased afterload (systolic blood pressure, patient-prosthesis mismatch, and high residual transvalvular gradient) and LVM regression: some authors support the hypothesis that these indexes have a negative correlation with reverse remodeling, whereas others do not find an association [4,6,7,8].

Reverse remodeling has been linked to a substantial impact on clinical outcomes. Lindman and her colleagues showed that in a population of patients with severe symptomatic aortic stenosis (AS) and severe LV hypertrophy, a greater decrease in LVM 30 days after TAVI was associated with lower hospitalization rates at one-year follow-up [9]; Chau and his colleagues described a 5–6% reduction in the risk of all-cause mortality, cardiovascular mortality, or re-hospitalization between 1 and 5 years for each 10% decrease in LVMi from baseline to one year. A greater LVMi regression was associated with a better quality of life at a two-year follow-up. Moreover, residual severe LV hypertrophy one-year post-TAVI was associated with a 71% increased risk of all-cause and cardiovascular mortality and an 89% increased risk of re-hospitalization [4].

In analogy with the LV, also the left atrium (LA) undergoes adaptive remodeling in AS. There is a functional connection between LV and LA: LV hypertrophy leads to LA decreased compliance and impaired relaxation with increased filling pressures; this, over time, induces LA dilation and progressive fibrosis [10]. Similar to the LV, structural and functional recovery occurs in the LA following the elimination of the aortic obstruction. This is reflected by decreased LA volume and increased peak atrial longitudinal strain (PALS) occurring relatively soon after TAVI, with the severity of AS being the key determinant of improvement [11]. In addition, LA speckle-tracking analysis has a predictive value: a PALS < 21% has been observed to predict major adverse cardiovascular events after TAVI [12].

### 2.2. Impact on LV Systolic Function

An early improvement in LV ejection fraction (EF) after TAVI occurs in approximately 50% of cases, but its prognostic significance remains controversial [13,14]. Several studies reported a lower risk of major adverse cardiac and cerebrovascular events and lower rates of one-year mortality, while other studies did not report an association between LV functional improvement and long-term outcomes at one-year follow-ups [13,14,15,16]. Greater LV mass, absence of hypertension, and a higher baseline transvalvular gradient are among the independent predictors of immediate post-procedural functional recovery [15]. Conversely, LV-EF ≤ 35%, a history of percutaneous coronary intervention, myocardial infarction, permanent pacemaker, and higher baseline EF are all predictors of lack of LV function improvement [13,14,16]. Data on the relationship between baseline moderate or severe AR with EF improvement are inconsistent [13,15].

Global longitudinal strain (GLS) has been proven to be one of the most effective methods for assessing subclinical LV dysfunction. Indeed, GLS proved to detect a significant improvement in LV function shortly after the procedure, even in the absence of significant changes in EF and irrespective of the THV type implanted [3]. Moreover, the lower the baseline LV systolic function, the better the improvement of both EF and GLS [16,17,18,19,20]. Patients with preserved LV-EF at baseline do not experience a decline or significant change in LV systolic function [17,20].

The analysis of layer-specific strain has provided further insight into the evaluation of LV function in AS patients undergoing TAVI, showing a more prominent impairment of the endocardial longitudinal strain in the advanced phases of the disease, with the occurrence of symptoms [21]. After TAVI, a significant improvement in all the myocardial layers has been reported, especially in the subendocardial one [22]. However, Cimino et al. showed a significant improvement in endocardial longitudinal strain early after TAVI only in patients with concentric hypertrophic remodeling [23].

**Figure 1:**
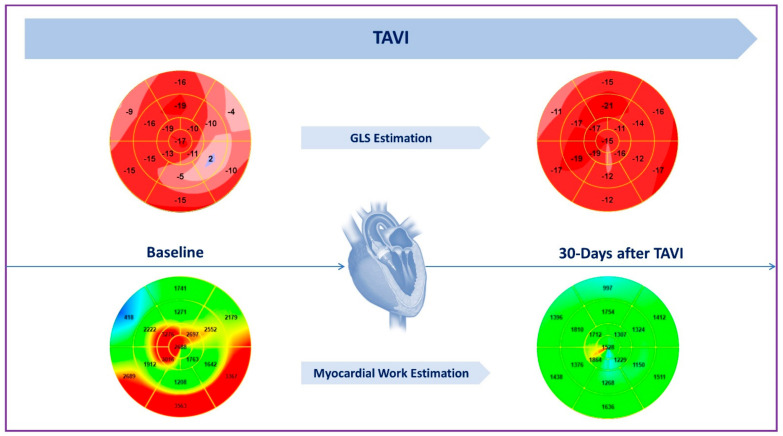
Global longitudinal strain (GLS), global work index (GWI), and global work efficiency (GWE) before and 30 days after TAVI.

Recently, noninvasive measurement of LV myocardial work (MW) has proven to be a valuable method for estimating myocardial performance in AS patients undergoing TAVI [24]. This tool incorporates echocardiographic strain data, cardiac events timing (from aortic and mitral valve opening and closure), and estimation of LV pressure (derived from systolic blood pressure) in order to derive global work index (GWI), global constructive work (GCW), global wasted work (GWW) and global work efficiency (GWE). In the specific setting of AS patients, LV pressure estimation is obtained by adding the mean aortic transvalvular gradient to the aortic systolic pressure. Jain et al. found that myocardial work indices decreased significantly following TAVI as a direct result of the acute relief of the aortic obstruction and decreased afterload. However, global work indices remained abnormal after the procedure, suggesting an incomplete recovery of the LV function after TAVI [25] (Figure 1). Moreover, lower values of GCW and GWI at baseline in AS patients have been associated with advanced stages of myocardial disease and with a worse prognosis even after aortic valve replacement [24].

In evaluating functional recovery during the follow-up, the presence of post-procedural AR plays a key role, as it can negatively impact LV remodeling and mimic an acute AR. Poulin et al. evaluated patients with new post-TAVI mild AR or moderate or severe AR (pre-existing or new) in contrast to non-important post-TAVI AR. The authors reported the absence of LV GLS improvement or positive remodeling in the presence of significant AR [20]. Moreover, moderate or severe AR was associated with a lack of LV end-diastolic volume index decrease after TAVI and represented the only independent correlate of survival at one-year follow-up in the study population of Sato and colleagues [26].

### 2.3. Impact on LV Diastolic Function

The LV hypertrophic response to the increased afterload results in the development of LV diastolic dysfunction (LVDD), which worsens progressively as the pressure overload persists. In a large cohort of AS patients undergoing TAVI, LVDD was found in about 70% of cases; among these patients, advancing stages of LVDD at baseline were independent predictors of all-cause mortality at one-year follow-up, with grade III as the strongest one. Moreover, one-year all-cause mortality increased progressively with worsening LVDD, with a higher risk among patients with grade III LVDD, irrespective of LV function. This incremental risk emerged as early as 30 days after the procedure, being mainly driven by cardiovascular death. Advanced stages of LVDD were also associated with prolonged hospitalization. After TAVI, no change in LVDD grade was documented in up to 50% of patients [27]. Blair et al. showed that there was a significant post-procedural improvement in several, but not all, diastolic parameters, including E-wave velocity, lateral e-velocity, E/lateral e, and left atrium volume index; they also demonstrated that improvement in LVDD grade was not significantly associated with improved outcomes after TAVI [28]. Conversely, Muratori et al. documented no association between baseline LVDD and survival, despite an improvement in LVDD during follow-up [29]. Moreover, in the presence of LVDD, the LV could not be able to increase compliance in response to an acute onset of paravalvular leak (PVL), leading to high elevation in LV end-diastolic pressures. There are discordant findings relative to the effect of LVDD in association with PVL on the risk of death: two studies reported an additive effect on one-year mortality [30,31], contrary to the results reported by Asami et al. showing that PVL did not further increase the risk of one-year mortality, beyond the effect of LVDD [27].

### 2.4. Impact on Mitral Regurgitation

MR often coexists with aortic stenosis: in patients undergoing TAVI, moderate or severe concomitant MR has a prevalence ranging from 11.5% to 36.8%, and degenerative MR is the underlying etiology in up to two-thirds of patients [32]. The impact of baseline MR on outcomes after TAVI has not been formally evaluated since severe MR represented an exclusion criterion in most of the main TAVI trials [33]. However, baseline moderate-to-severe MR increased the rates of re-hospitalization after 30 days as well as the rates of all-cause mortality after 30 days and one-year follow-up compared to non-mild MR, irrespective of the MR etiology [34,35,36,37]. In addition, primary MR showed to be associated with increased 30-day, two-year, and three-year mortality rates compared with secondary MR [37,38]. The rate of MR regression ranges from 47 to 78% as described in previous studies [32,35,36]. Mitral regurgitation tends to improve more likely and with a higher degree of regression in patients receiving a balloon-expandable valve [39]; indeed, it has been suggested that the longer stent frame of self-expanding valves (SEV) may anatomically and functionally interfere with the anterior mitral leaflet, especially in cases of lower implantation [40]. This finding was not confirmed in a subsequent study by Bedogni et al. including a large Core Valve cohort [41]. Furthermore, SEV implantation is associated with a higher incidence of PVL, left bundle-branch block, and pacemaker implantation that could lead to volume overload and adverse effects on LV remodeling, thus contributing to a reduced likelihood of MR improvement [39]. Furthermore, there are conflicting data on the impact of residual MR on outcomes after TAVI, with some but not all studies reporting a positive relationship between improved MR and higher survival rates [35,36,42,43,44]. Different mechanisms of post-procedural MR regression have been reported, mainly related to the hemodynamic changes occurring after the relief of the aortic obstruction: the reduced LV afterload decreases the trans-mitral pressure gradient. Consequently, reduced driving force results in MR grading improvement. Conversely, in the case of functional MR, the reduced LV-LA pressure gradient could sometimes lead to the persistence of MR due to a reduction in mitral valve closing forces [34]. On the other hand, reverse remodeling plays a role in functional MR improvement by reducing LV end-diastolic volumes and mitral valve tethering forces [45]. Mitral-aortic curtain compression, secondary to prosthetic valve deployment, has been proposed as an additional mechanism by which TAVI could impact mitral regurgitation severity [46,47]. Above all, besides the understanding of MR regression mechanisms, identifying clinical and echocardiographic predictors of MR improvement or MR persistence after TAVI is a matter of interest and this evaluation plays a key role in the pre-procedural phase [27,29,31,32,35,37,43,48] (Table 1). The identification of patients with the lowest or the highest probability of MR reduction is essential for defining the best management strategy and selecting the population that might more likely benefit from double-valve interventions [42]. Currently, a recent approach is to first perform TAVI, and, then, in the presence of suitable anatomical features, staged mitral percutaneous procedures can be scheduled for patients without significant MR regression [42]. In this context, MR re-assessment will represent one of the most critical steps of the echocardiographic follow-up program. Severity assessment should be performed by integrating different echocardiographic methods, with quantitative measurements always required in clinical practice.

## 3. TAVI and Right Heart

Cardiac damage in severe AS is not limited to the aortic valve and LV but is a systemic disease characterized by a significant alteration of the right heart as a result of ventricular interdependence. Severe AS is associated with chronic pressure overload of the LV, which elevates LA pressure, which is conveyed through the pulmonary vasculature and results in remodeling and dysfunction of the right heart [49,50]. This pathophysiological phenomenon presents with clinical and echocardiographic markers including pulmonary hypertension (PH), tricuspid regurgitation (TR), and right ventricular systolic dysfunction (RVSD) with a prevalence estimated to be about 30%, 20%, and 25%, respectively [51,52,53]. Their presence at baseline has been shown to have a negative prognostic impact on patients with AS undergoing TAVI with poor outcomes and more than a two-fold increased risk of cardiovascular death one year after TAVI, with a gradient of risk according to the recovery of RV dysfunction [49,54,55,56,57].

### 3.1. Impact on Right Ventricle Function

RV function has been evaluated using several parameters. Among them, the most used and clinically validated are tricuspid annular plane systolic excursion (TAPSE), tissue Doppler-derived tricuspid lateral annular systolic velocity (S′), percent RV fractional area change (FAC) and RV-EF [58,59]. According to current guidelines, RVSD is defined as at least one of the following: TAPSE < 17 mm, S′ < 9.5 cm/s, and RV FAC < 35% [60,61]. All these echocardiographic parameters have been shown to predict adverse outcomes and one-year mortality after TAVI [56].

A significant improvement in RV function has been described as early as 24 h after TAVI [62] and confirmed at mid-term follow-up with the increase in TAPSE and FAC values [63].

In a population of 144 patients undergoing TAVI, Leclercq et al. showed that, at six-month follow-up, at least one RVSD parameter (including TAPSE, S′, and FAC) significantly improved in 63.4% of patients, whereas a completely recovered normal RV function (with no TR) was achieved in 24.5% of patients [64]. RV recovery after TAVI seems to be closely related to the baseline RV systolic function, with no changes in the majority of patients with normal RV parameters at baseline, whereas in those with RVSD before TAVI, 50% experienced recovery of RV function during follow-up, showing better outcomes than those with persistent RVSD [57]. Similarly, an analysis of 226 patients undergoing TAVI with RVSD at baseline revealed that only 26% of patients with severe RVSD (defined as TAPSE < 10 mm) experienced an improvement in RV function, whereas patients with moderate RVSD (TAPSE between 10 and 16 mm) experienced an improvement more frequently (41%). In addition, this improvement is more likely to occur in the absence of atrial fibrillation, severe PH, or severe renal failure. Despite this, the improvement in RV function does not appear to be associated with improved survival [65]. Another essential issue is whether the RV function changes differently after TAVI vs. surgical aortic valve replacement (SAVR). A recent meta-analysis indicated that TAPSE and S′ were unchanged post-TAVI but decreased by 12 months following SAVR. Furthermore, both post-procedure TAPSE and △TAPSE were considerably better in the transfemoral-TAVI group compared to the SAVR group [54]. These data support the hypothesis that TAVI improves RV function and may be preferred to SAVR in patients with baseline RVSD. Hence, the RV function should be included in risk-scoring algorithms for patient selection.

### 3.2. Impact on Tricuspid Regurgitation and Pulmonary Hypertension

Echocardiography is the preferred tool for TR grading and PH evaluation [59,66,67]. In the majority of TAVI patients, TR is functional and the result of right-sided chamber remodeling (dilation, hypertrophy, and dysfunction). PH, a common finding in this patient population, may also contribute to worsening TR and differentiating adaptive RV remodeling with poor leaflet coaptation from advanced hemodynamic stress burdens that are secondary to long-standing AS. In the majority of patients undergoing TAVI, the suppression of pressure overload by TAVI reduces LV filling pressures in conjunction with a decrease in left atrial volume, pulmonary artery systolic pressure (PASP), and TR grading. TR evolution after TAVI is highly variable. Barbanti et al. showed that at 30 days after TAVI, TR response was unchanged in most of the patients (68%), while 15% experienced TR improvement and 17% had TR worsening, including 8% without significant TR before TAVI [52].

In the PARTNER II trial cohort B, among one-year survivors with nonsignificant TR at baseline, 19% had progression to significant TR [55]. Muraishi et al. showed a TR worsening from baseline mild or less TR to a moderate or severe grade in 87 patients (5.4%) with 3 independent predictors of TR progression: atrial fibrillation, transaortic mean pressure gradient < 40 mmHg on pre-TAVI TTE, and PASP > 40 mmHg [68].

The impact of TAVI on PH is another important aspect. In many AS patients, the pulmonary vasculature undergoes remodeling because of chronic pulmonary venous congestion; hence, PH is likely irreversible and persists following TAVI. Indeed, a significant decrease in PASP > 15 mmHg within one month of TAVI is observed in only a minority of patients (up to 35%) and more commonly in those without atrial fibrillation, severely depressed LV-EF, and severe MR [69]. On the other hand, a multicenter study showed significant changes in PASP after TAVI. Of 617 patients enrolled, 16% of patients without PH at baseline, PASP remained unchanged. In the remaining 84% of patients, a reversible PH was observed in 46%, with a change of PASP category of >1, from severe to mild-to-moderate or normal. In this cohort, LV-EF > 40%, baseline PASP > 46 mmHg, absence of moderate-to-severe TR, and logistic EuroSCORE < 25% were independent predictors of PASP reduction at discharge. Furthermore, the severity of PH at baseline does not predict post-procedural early or late mortality and therefore should not be considered a contraindication for TAVI [70].

Along the same line, Avvedimento et al. found a substantial decrease in PASP 30 days after TAVI [63]. A similar finding was reported from a two-center study: in this cohort, TAVI resulted in a significant and sustained reduction in PASP in the majority of survivors at follow-up after three months, which translated into a survival benefit compared to patients with persisting or new onset of severe PH [71].

In conclusion, the right heart, frequently described as the forgotten side of the heart, plays a key role in prognosis after TAVI, and its echocardiographic evaluation, in terms of RVSD, PH, and TR should be considered to stratify patients prior to TAVI and to evaluate cardiac damage evolution post-TAVI.

## 4. Endocarditis

Prosthetic valve endocarditis (PVE) is a rare but extremely serious complication that occurs in 0.3–2.3% of TAVI patients [72]. In the VARC-2 consensus document, PVE is defined as any of the following: fulfillment of the Duke criteria, evidence of abscess/paravalvular leak/pus/vegetation on reoperation or during autopsy [73,74].

Echocardiography plays a key role in PVE diagnosis, and it should be performed if PVE is suspected [74]. Bacterial proliferation can cause valve dehiscence, identified with TTE as PVL, with or without the rocking motion of the prosthesis [75]. Other echocardiographic findings included in Duke’s major criteria to diagnose endocarditis are vegetation, abscess, and pseudoaneurysm [74]. Endocarditis may also cause endocardium perforation, and consequently the communication of two cavities known as fistula, with color Doppler flow detected by TTE. If none of these findings is shown, TTE and/or transesophageal echocardiography (TEE) should be executed within five-seven days if clinical suspicion of endocarditis is high. Nonetheless, even in the case of TTE positivity or when it is not diagnostic, TEE should be performed to obtain a better characterization of the lesions and to exclude local complications [75]. The role of echocardiography is pivotal during the diagnostic phase as well as during follow-up. Uncomplicated PVE is conservatively managed and treated with medical targeted therapy; in this situation, TTE and/or TEE are performed to monitor vegetation dimensions, to rule out silent evolution, or in the case of clinical findings to suggest new complications (fever persistence, embolism, heart failure, etc.). Finally, at the end of medical therapy, TTE is repeated to assess valve morphology and function. Nonetheless, in the case of uncontrolled infection, refractory heart failure, and high embolic risk, patients should undergo surgical treatment. Noteworthy, intraoperative echocardiography is recommended in each of the aforementioned instances [75].

## 5. Thrombosis

Valve thrombosis is considered any thrombosis unrelated to infection, attached to or in close proximity to the valve that occludes part of the blood flow path, interfering with valve function or sufficiently large to warrant treatment [76]. It affects 0.6% of patients undergoing TAVI, particularly within the first year [77]. Various risk factors are involved in thrombus formation, including patient-related comorbidities (i.e., obesity, diabetes mellitus, chronic kidney disease) and valve implantation itself, which may cause endothelial damage and blood flow turbulences that represent predisposing factors for localized thrombogenesis. Clinical presentation of THV thrombosis is highly heterogeneous and includes patients with no symptoms, or conversely acute heart failure, embolic event, and most commonly progressive dyspnea [78]. Thus, echocardiography is the first step in assessing the presence of a thrombus. TTE should detect hypo-attenuated leaflet thickening (HALT) with relatively normal leaflet motion, HALT with reduced leaflet motion but normal gradients, and clinical valve thrombosis with elevated trans-prosthetic gradients [1]. Thrombus is identified as a mobile and globular mass with a soft echo density (comparable to that of the myocardium) which can cause, besides the above-mentioned abnormalities, central regurgitation, anomalous trans-prosthetic flow detected by color Doppler as aliasing, effective orifice area reduction [79]. Noteworthy, thrombosis may occur at the same time as pannus formation, determined by fibroblast proliferation and extra-cellular matrix deposition as a response to valve implantation. It is essential to differentiate between thrombus and pannus to guide patient management. Differential diagnoses can be obtained by means of imaging, firstly by means of echocardiography. Pannus is identified by TTE as a small mass with a bright echo density, different from the bigger and soft thrombus, and it tends to be fixed. Finally, it is less involved in leaflet motion reduction than thrombus and it is not responsive to anticoagulation, given its fibrotic nature [80,81].

## 6. Strengths and Pitfalls of Echocardiography after TAVI

Echocardiography is the key imaging modality for the evaluation of THVs, but there are some relevant challenges to recognize. For example, peak aortic jet velocity is highly flow dependent. This may lead to a significant overestimation in the case of a high-flow state. Any error in aortic jet velocity will inevitably result in an even larger overestimation of the mean transaortic pressure gradient. PVL severity evaluation can also be challenging. Therefore, measurements should always be interpreted in the clinical context as part of an integrative approach. In patients with poor acoustic windows, new onset severe PVL and valve dysfunction, or contradicting parameters, the use of other imaging modalities may be reasonable.

As well as with post-procedural complications (PVL, valve dysfunction, endocarditis, and thrombosis), TTE also has high sensitivity in detecting intraprocedural complications (prosthesis dislocation and limited anterior mitral leaflet mobility).

TEE performed during transcatheter structural cardiac interventions may result in greater complications than those performed in the nonoperative setting. In a study including 1249 patients requiring TEE guidance during TAVI, an incidence of 0.9% of overall complications was observed (among those, the most frequent were gastrointestinal bleeding, dysphagia, and odynophagia). Absolute contraindications to TEE include esophageal stricture, diverticulum, tumor, and recent esophageal or gastric surgery. Relative contraindications include cervical spine disease, hiatal hernia, coagulopathy, prior chest radiation, and facial or airway trauma [82,83].

## 7. Conclusions

Echocardiography is pivotal in the assessment of prosthesis function as well as cardiac function changes after TAVI. In particular, LVMi and PALS are useful tools recently implemented in clinal practice that are able to detect reverse remodeling early after TAVI. Similarly, GLS and MW estimation represent valuable methods for detecting an early recovery of LV systolic function. Moreover, TTE allows us to reassess MR severity after TAVI and to define the best management for patients with significant MR and persistent symptoms. In addition, RSVD, PH, and TR measurements enable us to evaluate the right heart function after TAVI and to estimate the patients’ prognoses. TEE is better than TTE at detecting endocarditis and valve thrombosis, two rare but potentially life-threatening complications. In conclusion, technical improvements and the implementation of new diagnostic tools have consolidated the main role of echocardiography in clinical outcomes evaluation after TAVI.

## Figures and Tables

**Table 1:** Suggested predictors of MR improvement and MR persistence after TAVI.

Factors Predicting MR Improvement	Factors Predicting MR Persistence or Worsening	
Functional etiology	Organic etiology	PPM
LV dilatation	Baseline severe MR	Use of SEV
Low ejection fraction	Permanent AF	Deep valve implantation
Coronary artery disease	Pulmonary hypertension	Calcified mitral valve disease
High transvalvular aortic gradient	Moderate or severe PVL	Mitral annular diameter > 35.5 mm

AF: atrial fibrillation; LV: left ventricle; MR: mitral regurgitation; PPM: patient-prosthesis mismatch; PVL: paravalvular leak; SEV: self-expandable valves.

## Data Availability

All data underlying this article will be shared on reasonable request to the corresponding author.

## References

[1] Vahanian A., Beyersdorf F., Praz F., Milojevic M., Baldus S., Bauersachs J., Capodanno D., Conradi L., De Bonis M., De Paulis R. (2022). 2021 ESC/EACTS Guidelines for the Management of Valvular Heart Disease. Eur. Heart J..

[2] Feghaly J., Das D., Oman Z., Smart S. (2021). Cardiac Structural Remodeling and Hemodynamic Patterns Following Transcatheter Aortic Valve Replacement. Cureus.

[3] Tsampasian V., Panoulas V., Jabbour R.J., Ruparelia N., Malik I.S., Hadjiloizou N., Frame A., Sen S., Sutaria N., Mikhail G.W. (2020). Left Ventricular Speckle Tracking Echocardiographic Evaluation before and after TAVI. Echo Res. Pract..

[4] Chau K.H., Douglas P.S., Pibarot P., Hahn R.T., Khalique O.K., Jaber W.A., Cremer P., Weissman N.J., Asch F.M., Zhang Y. (2020). Regression of Left Ventricular Mass After Transcatheter Aortic Valve Replacement. J. Am. Coll. Cardiol..

[5] Puls M., Beuthner B.E., Topci R., Vogelgesang A., Bleckmann A., Sitte M., Lange T., Backhaus S.J., Schuster A., Seidler T. (2020). Impact of Myocardial Fibrosis on Left Ventricular Remodelling, Recovery, and Outcome after Transcatheter Aortic Valve Implantation in Different Haemodynamic Subtypes of Severe Aortic Stenosis. Eur. Heart J..

[6] Pibarot P., Dumesnil J.G. (2000). Hemodynamic and Clinical Impact of Prosthesis-Patient Mismatch in the Aortic Valve Position and Its Prevention. J. Am. Coll. Cardiol..

[7] Rao V., Jamieson W.R., Ivanov J., Armstrong S., David T.E. (2000). Prosthesis-Patient Mismatch Affects Survival after Aortic Valve Replacement. Circulation.

[8] Tomoeda H., Ueda T., Teshima H., Arinaga K., Tayama K., Fukunaga S., Aoyagi S. (2010). Postoperative Left Ventricular Mass Regression after Aortic Valve Replacement for Aortic Stenosis. Ann. Thorac. Surg..

[9] Lindman B.R., Stewart W.J., Pibarot P., Hahn R.T., Otto C.M., Xu K., Devereux R.B., Weissman N.J., Enriquez-Sarano M., Wilson Szeto Y. (2014). Early Regression of Severe Left Ventricular Hypertrophy After Transcatheter Aortic Valve Replacement Is Associated With Decreased Hospitalizations. JACC Cardiovasc. Interv..

[10] Kampaktsis P.N., Kokkinidis D.G., Wong S.-C., Vavuranakis M., Skubas N.J., Devereux R.B. (2017). The Role and Clinical Implications of Diastolic Dysfunction in Aortic Stenosis. Heart.

[11] Lisi M., Pastore M.C., Fiorio A., Cameli M., Mandoli G.E., Righini F.M., Cavigli L., D’Ascenzi F., Focardi M., Rubboli A. (2022). Left Atrial Remodeling in Response to Aortic Valve Replacement: Pathophysiology and Myocardial Strain Analysis. Life.

[12] Galli E., Fournet M., Chabanne C., Lelong B., Leguerrier A., Flecher E., Mabo P., Donal E. (2016). Prognostic Value of Left Atrial Reservoir Function in Patients with Severe Aortic Stenosis: A 2D Speckle-Tracking Echocardiographic Study. Eur. Heart J. Cardiovasc. Imaging..

[13] Angelillis M., Giannini C., de Carlo M., Adamo M., Nardi M., Colombo A., Chieffo A., Bedogni F., Brambilla N., Tamburino C. (2017). Prognostic Significance of Change in the Left Ventricular Ejection Fraction After Transcatheter Aortic Valve Implantation in Patients With Severe Aortic Stenosis and Left Ventricular Dysfunction. Am. J. Cardiol..

[14] Elmariah S., Palacios I.F., McAndrew T., Hueter I., Inglessis I., Baker J.N., Kodali S., Leon M.B., Svensson L., Pibarot P. (2013). Outcomes of Transcatheter and Surgical Aortic Valve Replacement in High-Risk Patients with Aortic Stenosis and Left Ventricular Dysfunction: Results from the Placement of Aortic Transcatheter Valves (PARTNER) Trial (Cohort A). Circ. Cardiovasc. Interv..

[15] Jeong Y.J., Ahn J.-M., Kang D.-Y., Park H., Ko E., Kim H.J., Kim J.B., Choo S.J., Lee S.-A., Park S.-J. (2021). Incidence, Predictors, and Prognostic Impact of Immediate Improvement in Left Ventricular Systolic Function After Transcatheter Aortic Valve Implantation. Am. J. Cardiol..

[16] Kuneman J.H., Butcher S.C., Singh G.K., Wang X., Hirasawa K., van der Kley F., Leon M.B., Knuuti J., Pibarot P., Ajmone Marsan N. (2022). Prognostic Implications of Change in Left Ventricular Ejection Fraction After Transcatheter Aortic Valve Implantation. Am. J. Cardiol..

[17] Dimitriadis Z., Scholtz S., Ensminger S., Wiemer M., Fischbach T., Scholtz W., Piper C., Börgermann J., Bitter T., Horstkotte D. (2017). Left Ventricular Adaptation after TAVI Evaluated by Conventional and Speckle-Tracking Echocardiography. Int. J. Cardiol..

[18] Spethmann S., Baldenhofer G., Dreger H., Stüer K., Sanad W., Saghabalyan D., Müller E., Stangl V., Baumann G., Stangl K. (2014). Recovery of Left Ventricular and Left Atrial Mechanics in Various Entities of Aortic Stenosis 12 Months after TAVI. Eur. Heart. J. Cardiovasc. Imaging.

[19] Schueler R., Sinning J.-M., Momcilovic D., Weber M., Ghanem A., Werner N., Nickenig G., Grube E., Hammerstingl C. (2012). Three-Dimensional Speckle-Tracking Analysis of Left Ventricular Function after Transcatheter Aortic Valve Implantation. J. Am. Soc. Echocardiogr..

[20] Poulin F., Carasso S., Horlick E.M., Rakowski H., Lim K.-D., Finn H., Feindel C.M., Greutmann M., Osten M.D., Cusimano R.J. (2014). Recovery of Left Ventricular Mechanics after Transcatheter Aortic Valve Implantation: Effects of Baseline Ventricular Function and Postprocedural Aortic Regurgitation. J. Am. Soc. Echocardiogr..

[21] Ilardi F., Marchetta S., Martinez C., Sprynger M., Ancion A., Manganaro R., Sugimoto T., Tsugu T., Postolache A., Piette C. (2020). Impact of Aortic Stenosis on Layer-Specific Longitudinal Strain: Relationship with Symptoms and Outcome. Eur. Heart J. Cardiovasc. Imaging.

[22] Shiino K., Yamada A., Scalia G.M., Putrino A., Chamberlain R., Poon K., Walters D.L., Chan J. (2019). Early Changes of Myocardial Function after Transcatheter Aortic Valve Implantation Using Multilayer Strain Speckle Tracking Echocardiography. Am. J. Cardiol..

[23] Cimino S., Monosilio S., Luongo F., Neccia M., Birtolo L.I., Salvi N., Filomena D., Mancone M., Fedele F., Agati L. (2021). Myocardial Contractility Recovery Following Acute Pressure Unloading after Transcatheter Aortic Valve Intervention (TAVI) in Patients with Severe Aortic Stenosis and Different Left Ventricular Geometry: A Multilayer Longitudinal Strain Echocardiographicanalysis. Int. J. Cardiovasc. Imaging.

[24] Ilardi F., Postolache A., Dulgheru R., Trung M.-L.N., de Marneffe N., Sugimoto T., Go Y.Y., Oury C., Esposito G., Lancellotti P. (2022). Prognostic Value of Non-Invasive Global Myocardial Work in Asymptomatic Aortic Stenosis. J. Clin. Med..

[25] Jain R., Bajwa T., Roemer S., Huisheree H., Allaqaband S.Q., Kroboth S., Perez Moreno A.C., Tajik A.J., Khandheria B.K. (2021). Myocardial Work Assessment in Severe Aortic Stenosis Undergoing Transcatheter Aortic Valve Replacement. Eur. Heart J. Cardiovasc. Imaging.

[26] Sato K., Kumar A., Jones B.M., Mick S.L., Krishnaswamy A., Grimm R.A., Desai M.Y., Griffin B.P., Rodriguez L.L., Kapadia S.R. (2017). Reversibility of Cardiac Function Predicts Outcome after Transcatheter Aortic Valve Replacement in Patients with Severe Aortic Stenosis. J. Am. Heart Assoc..

[27] Asami M., Lanz J., Stortecky S., Räber L., Franzone A., Heg D., Hunziker L., Roost E., Siontis G.C., Valgimigli M. (2018). The Impact of Left Ventricular Diastolic Dysfunction on Clinical Outcomes after Transcatheter Aortic Valve Replacement. JACC Cardiovasc. Interv..

[28] Blair J.E.A., Atri P., Friedman J.L., Thomas J.D., Brummel K., Sweis R.N., Mikati I., Malaisrie S.C., Davidson C.J., Flaherty J.D. (2017). Diastolic Function and Transcatheter Aortic Valve Replacement. J. Am. Soc. Echocardiogr..

[29] Muratori M., Fusini L., Tamborini G., Gripari P., Delgado V., Marsan N.A., Ghulam Ali S., Barbier P., Bartorelli A.L., Alamanni F. (2016). Sustained Favourable Haemodynamics 1 Year after TAVI: Improvement in NYHA Functional Class Related to Improvement of Left Ventricular Diastolic Function. Eur. Heart J. Cardiovasc. Imaging.

[30] Kampaktsis P.N., Bang C.N., Chiu Wong S., Skubas N.J., Singh H., Voudris K., Baduashvili A., Pastella K., Swaminathan R.V., Kaple R.K. (2017). Prognostic Importance of Diastolic Dysfunction in Relation to Post Procedural Aortic Insufficiency in Patients Undergoing Transcatheter Aortic Valve Replacement. Catheter. Cardiovasc. Interv..

[31] Conte L., Fabiani I., Pugliese N.R., Giannini C., La Carruba S., Angelillis M., Spontoni P., De Carlo M., Petronio A.S., Di Bello V. (2017). Left Ventricular Stiffness Predicts Outcome in Patients with Severe Aortic Stenosis Undergoing Transcatheter Aortic Valve Implantation. Echocardiography.

[32] Khan F., Okuno T., Malebranche D., Lanz J., Praz F., Stortecky S., Windecker S., Pilgrim T. (2020). Transcatheter Aortic Valve Replacement in Patients With Multivalvular Heart Disease. JACC Cardiovasc. Interv..

[33] Sengupta A., Biswas M., Zaid S., Alexis S.L., Tang G.H.L. (2020). Effect & Implications of Transcatheter Aortic Valve Replacement on Concomitant Functional Mitral Regurgitation. Struct. Heart.

[34] Nombela-Franco L., Ribeiro H.B., Urena M., Allende R., Amat-Santos I., DeLarochellière R., Dumont E., Doyle D., DeLarochellière H., Laflamme J. (2014). Significant Mitral Regurgitation Left Untreated at the Time of Aortic Valve Replacement. J. Am. Coll. Cardiol..

[35] Chakravarty T., van Belle E., Jilaihawi H., Noheria A., Testa L., Bedogni F., Rück A., Barbanti M., Toggweiler S., Thomas M. (2015). Meta-Analysis of the Impact of Mitral Regurgitation on Outcomes after Transcatheter Aortic Valve Implantation. Am. J. Cardiol..

[36] Cortés C., Amat-Santos I.J., Nombela-Franco L., Muñoz-Garcia A.J., Gutiérrez-Ibanes E., de La J.M., Hernandez T., Córdoba-Soriano J.G., Jimenez-Quevedo P., Hernández-García J.M. (2016). Mitral Regurgitation After Transcatheter Aortic Valve Replacement Prognosis, Imaging Predictors, and Potential Management. JACC Cardiovasc. Interv..

[37] Muratori M., Fusini L., Tamborini G., Ghulam Ali S., Gripari P., Fabbiocchi F., Salvi L., Trabattoni P., Roberto M., Agrifoglio M. (2020). Mitral Valve Regurgitation in Patients Undergoing TAVI: Impact of Severity and Etiology on Clinical Outcome. Int. J. Cardiol..

[38] Vollenbroich R., Stortecky S., Praz F., Lanz J., Franzone A., Zuk K., Heg D., Valgimigli M., O’Sullivan C.J., Heinisch C. (2017). The Impact of Functional vs. Degenerative Mitral Regurgitation on Clinical Outcomes among Patients Undergoing Transcatheter Aortic Valve Implantation. Am. Heart J..

[39] Nombela-Franco L., Eltchaninoff H., Zahn R., Testa L., Leon M.B., Trillo-Nouche R., Donofrio A., Smith C.R., Webb J., Bleiziffer S. (2015). Clinical Impact and Evolution of Mitral Regurgitation Following Transcatheter Aortic Valve Replacement: A Meta-Analysis. Heart.

[40] De Chiara B., Moreo A., de Marco F., Musca F., Oreglia J., Lobiati E., Bruschi G., Belli O., Mauri F., Klugmann S. (2011). Influence of CoreValve ReValving System Implantation on Mitral Valve Function: An Echocardiographic Study in Selected Patients. Catheter. Cardiovasc. Interv..

[41] Bedogni F., Latib A., de Marco F., Agnifili M., Oreglia J., Pizzocri S., Latini R.A., Lanotte S., Petronio A.S., de Carlo M. (2013). Interplay between Mitral Regurgitation and Transcatheter Aortic Valve Replacement with the CoreValve Revalving System: A Multicenter Registry. Circulation.

[42] Witberg G., Codner P., Landes U., Schwartzenberg S., Barbanti M., Valvo R., de Backer O., Ooms J.F., Islas F., Marroquin L. (2021). Effect of Transcatheter Aortic Valve Replacement on Concomitant Mitral Regurgitation and Its Impact on Mortality. JACC Cardiovasc. Interv..

[43] Mavromatis K., Thourani V.H., Stebbins A., Vemulapalli S., Devireddy C., Guyton R.A., Matsouaka R., Ghasemzadeh N., Block P.C., Leshnower B.G. (2017). Transcatheter Aortic Valve Replacement in Patients With Aortic Stenosis and Mitral Regurgitation. Ann. Thorac. Surg..

[44] Mauri V., Körber M.I., Kuhn E., Schmidt T., Frerker C., Wahlers T., Rudolph T.K., Baldus S., Adam M., ten Freyhaus H. (2020). Prognosis of Persistent Mitral Regurgitation in Patients Undergoing Transcatheter Aortic Valve Replacement. Clin. Res. Cardiol..

[45] Unger P., Plein D., van Camp G., Cosyns B., Pasquet A., Henrard V., de Cannière D., Melot C., Piérard L.A., Lancellotti P. (2008). Effects of Valve Replacement for Aortic Stenosis on Mitral Regurgitation. Am. J. Cardiol..

[46] Caballero A., Mao W., McKay R., Sun W. (2019). The Impact of Balloon-Expandable Transcatheter Aortic Valve Replacement on Concomitant Mitral Regurgitation: A Comprehensive Computational Analysis. J. R. Soc. Interface.

[47] Caballero A., Mao W., McKay R., Sun W. (2020). The Impact of Self-Expandable Transcatheter Aortic Valve Replacement on Concomitant Functional Mitral Regurgitation: A Comprehensive Engineering Analysis. Struct. Heart.

[48] Boerlage-van Dijk K., Wiegerinck E.M.A., Takama T., Koch K.T., Vis M.M., de Mol B.A.J.M., Piek J.J., Bouma B.J., Baan J. (2016). Mitral Regurgitation Prior to Transcatheter Aortic Valve Implantation Influences Survival but Not Symptoms. Int. J. Cardiol..

[49] Asami M., Stortecky S., Praz F., Lanz J., Räber L., Franzone A., Piccolo R., Siontis G.C.M., Heg D., Valgimigli M. (2019). Prognostic Value of Right Ventricular Dysfunction on Clinical Outcomes After Transcatheter Aortic Valve Replacement. JACC Cardiovasc. Imaging.

[50] Eleid M.F., Padang R., Pislaru S.V., Greason K.L., Crestanello J., Nkomo V.T., Pellikka P.A., Jentzer J.C., Gulati R., Sandhu G.S. (2019). Effect of Transcatheter Aortic Valve Replacement on Right Ventricular–Pulmonary Artery Coupling. JACC Cardiovasc. Interv..

[51] Hutter A., Bleiziffer S., Richter V., Opitz A., Hettich I., Mazzitelli D., Ruge H., Lange R. (2013). Transcatheter Aortic Valve Implantation in Patients with Concomitant Mitral and Tricuspid Regurgitation. Ann. Thorac. Surg..

[52] Barbanti M., Binder R.K., Dvir D., Tan J., Freeman M., Thompson C.R., Cheung A., Wood D.A., Leipsic J., Webb J.G. (2015). Prevalence and Impact of Preoperative Moderate/Severe Tricuspid Regurgitation on Patients Undergoing Transcatheter Aortic Valve Replacement. Catheter. Cardiovasc. Interv..

[53] Galli E., Guirette Y., Feneon D., Daudin M., Fournet M., Leguerrier A., Flecher E., Mabo P., Donal E. (2015). Prevalence and Prognostic Value of Right Ventricular Dysfunction in Severe Aortic Stenosis. Eur. Heart J. Cardiovasc. Imaging.

[54] Ren B., Spitzer E., Geleijnse M.L., Zijlstra F., de Jaegere P.P.T., van Mieghem N.M., Tijssen J.G. (2018). Right Ventricular Systolic Function in Patients Undergoing Transcatheter Aortic Valve Implantation: A Systematic Review and Meta-Analysis. Int. J. Cardiol..

[55] Lindman B.R., Maniar H.S., Jaber W.A., Lerakis S., Mack M.J., Suri R.M., Thourani V.H., Babaliaros V., Kereiakes D.J., Whisenant B. (2015). Effect of Tricuspid Regurgitation and the Right Heart on Survival after Transcatheter Aortic Valve Replacement: Insights from the Placement of Aortic Transcatheter Valves II Inoperable Cohort. Circ. Cardiovasc. Interv..

[56] Grevious S.N., Fernandes M.F., Annor A.K., Ibrahim M., Saint Croix G.R., de Marchena E., Cohen M.G., Alfonso C.E. (2020). Prognostic Assessment of Right Ventricular Systolic Dysfunction on Post–Transcatheter Aortic Valve Replacement Short-Term Outcomes: Systematic Review and Meta-Analysis. J. Am. Heart Assoc..

[57] Poch F., Thalmann R., Olbrich I., Fellner C., Stundl A., Barthel P., Bradaric C., Laugwitz K.L., Kupatt C., Ledwoch J. (2021). Changes of Right Ventricular Function After Transcatheter Aortic Valve Replacement and Association With Outcomes. J. Card. Fail..

[58] Lang R.M., Bierig M., Devereux R.B., Flachskampf F.A., Foster E., Pellikka P.A., Picard M.H., Roman M.J., Seward J., Shanewise J.S. (2005). Recommendations for Chamber Quantification: A Report from the American Society of Echocardiography’s Guidelines and Standards Committee and the Chamber Quantification Writing Group, Developed in Conjunction with the European Association of Echocardiography, a Branch of the European Society of Cardiology. J. Am. Soc. Echocardiogr..

[59] Rudski L.G., Lai W.W., Afilalo J., Hua L., Handschumacher M.D., Chandrasekaran K., Solomon S.D., Louie E.K., Schiller N.B. (2010). Guidelines for the Echocardiographic Assessment of the Right Heart in Adults: A Report from the American Society of Echocardiography Endorsed by the European Association of Echocardiography, a Registered Branch of the European Society of Cardiology, and the Canadian Society of Echocardiography. J. Am. Soc. Echocardiogr..

[60] Harjola V.P., Mebazaa A., Čelutkiene J., Bettex D., Bueno H., Chioncel O., Crespo-Leiro M.G., Falk V., Filippatos G., Gibbs S. (2016). Contemporary Management of Acute Right Ventricular Failure: A Statement from the Heart Failure Association and the Working Group on Pulmonary Circulation and Right Ventricular Function of the European Society of Cardiology. Eur. J. Heart Fail..

[61] Lang R.M., Badano L.P., Victor M.A., Afilalo J., Armstrong A., Ernande L., Flachskampf F.A., Foster E., Goldstein S.A., Kuznetsova T. (2015). Recommendations for Cardiac Chamber Quantification by Echocardiography in Adults: An Update from the American Society of Echocardiography and the European Association of Cardiovascular Imaging. J. Am. Soc. Echocardiogr..

[62] Ayhan H., Durmaz T., Keleş T., Sari C., Aslan A.N., Kasapkara H.A., Bozkurt E. (2014). Improvement of Right Ventricular Function with Transcatheter Aortic Valve Implantation. Scand. Cardiovasc. J..

[63] Avvedimento M., Franzone A., Leone A., Piccolo R., Castiello D.S., Ilardi F., Mariani A., Esposito R., Iapicca C., Angellotti D. (2021). Extent of Cardiac Damage and Mortality in Patients Undergoing Transcatheter Aortic Valve Implantation. J. Clin. Med..

[64] Leclercq F., Lorca L., Agullo A., Bouchdoug K., Macia J.C., Delseny D., Roubille F., Gandet T., Lattuca B., Robert P. (2022). Evolution of Right Ventricular Dysfunction and Tricuspid Regurgitation after TAVI: A Prospective Study. Int. J. Cardiol..

[65] Testa L., Latib A., de Marco F., de Carlo M., Fiorina C., Barbanti M., Montone R.A., Agnifili M., Petronio A.S., Ettori F. (2016). The Failing Right Heart: Implications and Evolution in High-Risk Patients Undergoing Transcatheter Aortic Valve Implantation. EuroIntervention.

[66] Hahn R.T., Thomas J.D., Khalique O.K., Cavalcante J.L., Praz F., Zoghbi W.A. (2019). Imaging Assessment of Tricuspid Regurgitation Severity. JACC Cardiovasc. Imaging.

[67] Parasuraman S., Walker S., Loudon B.L., Gollop N.D., Wilson A.M., Lowery C., Frenneaux M.P. (2016). Assessment of Pulmonary Artery Pressure by Echocardiography—A Comprehensive Review. Int. J. Cardiol. Heart Vasc..

[68] Muraishi M., Tabata M., Shibayama K., Ito J., Shigetomi K., Obunai K., Watanabe H., Yamamoto M., Watanabe Y., Naganuma T. (2022). Late Progression of Tricuspid Regurgitation After Transcatheter Aortic Valve Replacement. J. Soc. Cardiovasc. Angiogr. Interv..

[69] Testa L., Latib A., de Marco F., de Carlo M., Fiorina C., Montone R., Agnifili M., Barbanti M., Petronio A.S., Zoccai G.B. (2016). Persistence of Severe Pulmonary Hypertension after Transcatheter Aortic Valve Replacement: Incidence and Prognostic Impact. Circ. Cardiovasc. Interv..

[70] Alushi B., Beckhoff F., Leistner D., Franz M., Reinthaler M., Stähli B.E., Morguet A., Figulla H.R., Doenst T., Maisano F. (2019). Pulmonary Hypertension in Patients With Severe Aortic Stenosis: Prognostic Impact After Transcatheter Aortic Valve Replacement: Pulmonary Hypertension in Patients Undergoing TAVR. JACC Cardiovasc. Imaging.

[71] Sinning J.M., Hammerstingl C., Chin D., Ghanem A., Schueler R., Sedaghat A., Bence J., Spyt T., Werner N., Kovac J. (2014). Decrease of Pulmonary Hypertension Impacts on Prognosis after Transcatheter Aortic Valve Replacement. EuroIntervention.

[72] Eisen A., Shapira Y., Sagie A., Kornowski R. (2012). Infective Endocarditis in the Transcatheter Aortic Valve Replacement Era: Comprehensive Review of a Rare Complication. Clin. Cardiol..

[73] Kappetein A.P., Head S.J., Généreux P., Piazza N., van Mieghem N.M., Blackstone E.H., Brott T.G., Cohen D.J., Cutlip D.E., van Es G.A. (2012). Updated Standardized Endpoint Definitions for Transcatheter Aortic Valve Implantation: The Valve Academic Research Consortium-2 Consensus Document. Eur. Heart J..

[74] Durack D.T., Phil D., Lukes A.S., Bright D.K., Service E. (1994). New Criteria for Diagnosis of Infective Endocarditis: Utilization of Specific Echocardiographic Findings. Am. J. Med..

[75] Habib G., Lancellotti P., Antunes M.J., Bongiorni M.G., Casalta J.P., del Zotti F., Dulgheru R., el Khoury G., Erba P.A., Iung B. (2015). 2015 ESC Guidelines for the Management of Infective Endocarditis. Eur. Heart J..

[76] Akins C.W., Miller D.C., Turina M.I., Kouchoukos N.T., Blackstone E.H., Grunkemeier G.L., Takkenberg J.J.M., David T.E., Butchart E.G., Adams D.H. (2008). Guidelines for Reporting Mortality and Morbidity After Cardiac Valve Interventions. Ann. Thorac. Surg..

[77] Latib A., Naganuma T., Abdel-Wahab M., Danenberg H., Cota L., Barbanti M., Baumgartner H., Finkelstein A., LeGrand V., de Lezo J.S. (2015). Treatment and Clinical Outcomes of Transcatheter Heart Valve Thrombosis. Circ. Cardiovasc. Interv..

[78] Dangas G.D., Weitz J.I., Giustino G., Makkar R., Mehran R. (2016). Prosthetic Heart Valve Thrombosis. J. Am. Coll. Cardiol..

[79] Roudaut R., Serri K., Lafitte S. (2007). Thrombosis of Prosthetic Heart Valves: Diagnosis and Therapeutic Considerations. Heart.

[80] Zoghbi W.A., Chambers J.B., Dumesnil J.G., Foster E., Gottdiener J.S., Grayburn P.A., Khandheria B.K., Levine R.A., Marx G.R., Miller F.A. (2009). Recommendations for Evaluation of Prosthetic Valves With Echocardiography and Doppler Ultrasound. A Report From the American Society of Echocardiography’s Guidelines and Standards Committee and the Task Force on Prosthetic Valves, Developed in Conjunction With the American College of Cardiology Cardiovascular Imaging Committee, Cardiac Imaging Committee of the American Heart Association. J. Am. Soc. Echocardiogr..

[81] Lancellotti P., Pibarot P., Chambers J., Edvardsen T., Delgado V., Dulgheru R., Pepi M., Cosyns B., Dweck M.R., Garbi M. (2016). Recommendations for the Imaging Assessment of Prosthetic Heart Valves: A Report from the European Association of Cardiovascular Imaging Endorsed by the Chinese Society of Echocardiography, the Inter-American Society of Echocardiography, and the Brazilian Department of Cardiovascular Imaging. Eur. Heart J. Cardiovasc. Imaging.

[82] Freitas-Ferraz A.B., Rodés-Cabau J., Junquera Vega L., Beaudoin J., O’Connor K., Turgeon P.Y., Paradis J.-M., Ferreira-Neto A., Asmarats L., Champagne J. (2020). Transesophageal Echocardiography Complications Associated with Interventional Cardiology Procedures. Am. Heart J..

[83] Hasnie A.A., Parcha V., Hawi R., Trump M., Shetty N.S., Ahmed M.I., Booker O.J., Arora P., Arora G. (2023). Complications Associated With Transesophageal Echocardiography in Transcatheter Structural Cardiac Interventions. J. Am. Soc. Echocardiogr..

